# Can Insects Develop Resistance to Insect Pathogenic Fungi?

**DOI:** 10.1371/journal.pone.0060248

**Published:** 2013-04-01

**Authors:** Ivan M. Dubovskiy, Miranda M. A. Whitten, Olga N. Yaroslavtseva, Carolyn Greig, Vadim Y. Kryukov, Ekaterina V. Grizanova, Krishnendu Mukherjee, Andreas Vilcinskas, Viktor V. Glupov, Tariq M. Butt

**Affiliations:** 1 Institute of Systematics and Ecology of Animals, Siberian Branch of Russian Academy of Sciences, Novosibirsk, Russia; 2 Institute of Life Sciences, College of Medicine, Swansea University, Swansea, United Kingdom; 3 Department of Biosciences, College of Science, Swansea University, Swansea, United Kingdom; 4 Institut für Phytopathologie und Angewandte Zoologie, Abteilung Angewandte Entomologie, Gießen, Germany; Ecole Normale Supérieur de Lyon, France

## Abstract

Microevolutionary adaptations and mechanisms of fungal pathogen resistance were explored in a melanic population of the Greater wax moth, *Galleria mellonella*. Under constant selective pressure from the insect pathogenic fungus *Beauveria bassiana*, 25^th^ generation larvae exhibited significantly enhanced resistance, which was specific to this pathogen and not to another insect pathogenic fungus, *Metarhizium anisopliae.* Defense and stress management strategies of selected (resistant) and non-selected (susceptible) insect lines were compared to uncover mechanisms underpinning resistance, and the possible cost of those survival strategies. We hypothesize that the insects developed a transgenerationally primed resistance to the fungus *B. bassiana*, a costly trait that was achieved not by compromising life-history traits but rather by prioritizing and re-allocating pathogen-species-specific augmentations to integumental front-line defenses that are most likely to be encountered by invading fungi. Specifically during *B. bassiana* infection, systemic immune defenses are suppressed in favour of a more limited but targeted repertoire of enhanced responses in the cuticle and epidermis of the integument (e.g. expression of the fungal enzyme inhibitor IMPI, and cuticular phenoloxidase activity). A range of putative stress-management factors (e.g. antioxidants) is also activated during the specific response of selected insects to *B. bassiana* but not *M. anisopliae.* This too occurs primarily in the integument, and probably contributes to antifungal defense and/or helps ameliorate the damage inflicted by the fungus or the host’s own immune responses.

## Introduction

Insects are predominantly dependent upon cuticular, humoral and cellular defenses to resist fungal pathogens. The cuticle is the primary and possibly the most important barrier to fungal infection. Fungistatic fatty acids, phenoloxidases and melanins can help resist penetration of the cuticle [1]. If the pathogen is able to breach the cuticle, it then has to contend with the host’s humoral and cellular defenses [2]. The latter consist of hemocytes, which will participate in wound healing and encapsulate fungal elements too large to be phagocytosed. Key humoral elements include phenoloxidase, reactive oxygen species and antimicrobial peptides [3]. Phenoloxidase synthesizes melanin, a highly fungitoxic compound which is deposited on the fungal surface and may block fungal development by encapsulating the pathogen in a melanic sheath [4], [5]. A wide range of antimicrobial peptides (AMPs) have been reported in insects with most of them showing antibacterial activity while relatively few have been identified with antifungal activity [6], [7], [8], [9]. Some AMPs appear to be peculiar to specific insect species while others have been reported in several insect species suggesting that some share a common ancestry while others have evolved independently [10].

Insect pathogenic fungi, of which there are over 700 species, have evolved to counter the host’s defenses using a combination of enzymes to penetrate the cuticle and access the nutrient rich contents of the hemocoel [11]. During the colonization phase, the pathogen will produce a wide range of secondary metabolites that may suppress the host’s immune system [12], [13] and concomitantly prevent secondary infections. The type and quantity of metabolite produced *in vivo* is dependent upon the host species and fungal strain [14], [15]. Fungi have evolved additional strategies to evade the host immune defenses. For example, hyphal bodies of *Metarhizium anisopliae,* during colonization of the hemocoel, will mask the cell wall by coating it with collagen-like material [16]. The integument, therefore, may represent the best opportunity for the insect host to detect and incapacitate the fungal pathogen.

There is much interest in insect pathogenic fungi because they are considered to offer an environmentally friendly alternative to chemical pesticides, which have been withdrawn or to which pests have developed resistance. Strains of fungi have been identified that kill both crop pests as well as pests of veterinary and medical importance such as ticks, midges and mosquitoes [17], [18], [19]. Of these fungi, the hypocrealean ascomycetes *M. anisopliae* and *Beauveria bassiana* are the best characterized and the most widely used in biological control programs. About thirteen species or sub-species of both fungi have been formulated and registered as mycoinsecticides or mycoacaricides [20]. Of 171 products reviewed by [20] ca. 68% were products based on *Beauveria* and *Metarhizium.* The use of these fungi is expected to increase following new EU legislation particularly EC regulation 1107/2009 and Directive 2009/128/EC which make it obligatory for EU member states to implement the principles of Integrated Pest Management with priority to be given to non-chemical methods of pest control.

The increased use of insect pathogenic fungi raises the question: will insects develop resistance to these agents in the same way that they developed resistance to chemical pesticides? Resistance is a major concern of producers of these fungi as biological control agents (BCAs) as they need to recoup development costs, but also of end users who are trapped between a diminishing number of chemical pesticides and the lack of safe alternatives.

Insect species, populations and intrapopulation groups differ in their susceptibility to fungal infections, which possibly reflects adaptation of the immune defenses to local conditions. Differences may be linked to a wide range of factors including the presence or absence of symbionts, density-linked melanism or genetic variation [21], [22], [23], [24], [25], [26]. The darkened cuticle of melanic insects intrinsically confers some degree of resistance to insect pathogenic fungi [27], [28], [29].

Studying induced resistance poses many challenges because of the large number of parameters that need to be considered, often involving complex physiological responses. One approach is to select for increased resistance to a natural pathogen under specific laboratory conditions, then to identify correlated responses to selection and associated cost-benefits. Kraaijeveld and Godfray [30] used such an approach to explore the evolution of resistance to *B. bassiana* in *Drosophila melanogaster* and found no evidence of resistance after fifteen generations of selection. The authors did note increased late-life fecundity in selected lines suggesting evolved tolerance of fungal infection. In the absence of fungal infection, these selected flies had lower fitness than control flies indicating a trade off with increased tolerance [30]. In another study, exposure of *D. melanogaster* over twenty six generations to an antagonistic (but not true insect-pathogenic) fungus *Aspergillus nidulans*, resulted in selected lines with no increase in resistance but a reduced sensitivity to sterigmatocystin, a toxin produced by this fungus [31]. The underlying mechanism(s) for the increased toxin specific tolerance is unclear.

In this paper, we report an artificial selection experiment designed to explore the evolution of resistance in a melanic morph of the Greater wax moth, *Galleria mellonella,* to natural topical infection by *B. bassiana.* We ask whether this morph possesses additional traits that allow increased resistance to evolve, and, if so, at what cost. We recognise that resistance is not entirely mediated by an immune response and may involve multiple factors including stress management, energy re-allocation and tissue specific resource distribution. We therefore compared several of these parameters in *G. mellonella* larvae selected for resistance *versus* a non-selected line. In addition, we tested specificity by measuring the insects’ responses to another pathogenic fungus, *M. anisopliae*.

## Results

### Selection and Insect Survival following *B. bassiana* and *M. anisopliae* Topical Treatment

**Figure 1:**
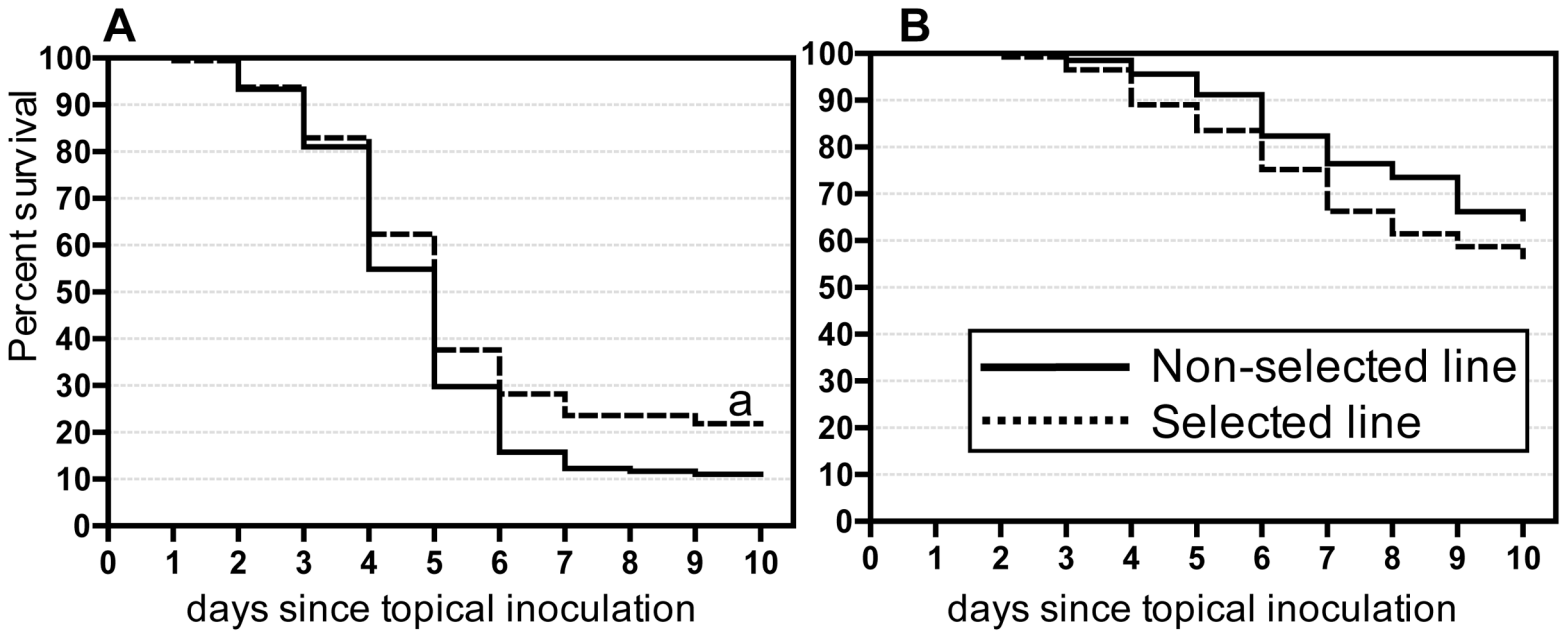
Susceptibility of insects selected by B. bassiana to the fungal infections. Mortality rate of selected line and non-selected line of *G. mellonella* larvae following topical treatment with the fungus *B. bassiana* (A) and *M.anisopliae* (B). (a-P<0.01 compared with non-selected line larvae. n  = 140–190 per line per treatment).

Larvae of a melanic phenotype of the Greater wax moth, *Galleria mellonella,* were selected for resistance to *B. bassiana* over 25 generations (Table S1). Survival assays conducted on cohorts of the selected (S) and non-selected (NS) lines revealed significantly increased resistance (survival times) of the 25^th^ generation of the S line to *B. bassiana* compared with the NS line (Figure 1A); (P = 0.01). Importantly, this S line did not show statistically significant cross-resistance to *M. anisopliae* than the NS insects (Figure 1B). The defense responses of the S and NS lines to *B. bassiana* and *M. anisopliae* were investigated further to elucidate the underlying mechanisms for the increased resistance at species level.

### Cuticular, Cellular and Humoral Immune Defenses following Topical Inoculation

**Figure 2:**
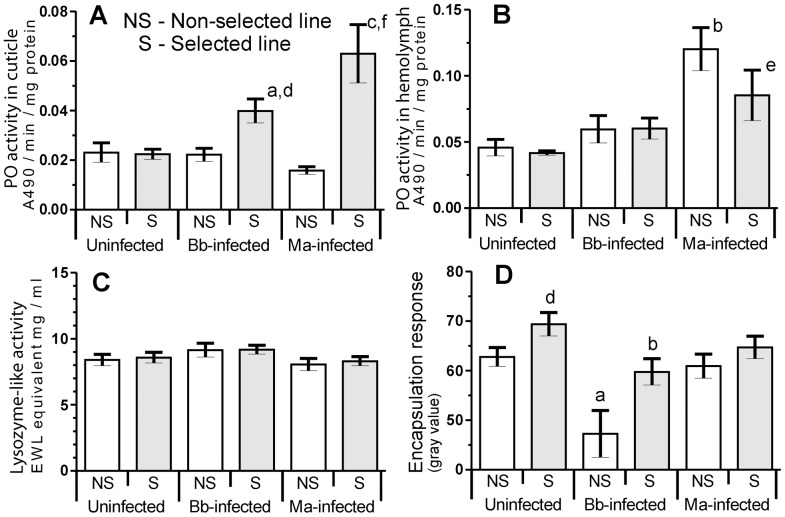
Immune function of insects selected by *B. bassiana* under infections. Cuticular phenoloxidase (PO) activity (A), hemolymph phenoloxidase (B), lysozyme-like (C) activity and encapsulation responses (D) in hemolymph of *G. mellonella* larvae from non-selected (NS) and selected (S) lines at 24 h following topical application of *B. bassiana* (Bb) and *M. anisopliae* (Ma) (data presented as mean +/− SEM; a-P<0.05, b-P<0.01, c-P<0.001 compared with uninfected larvae from the same line; d-P<0.05 e-P<0.01 f-P<0.001 compared with NS larvae with the same treatment).

The NS and S larvae had identical levels of basal (uninfected) PO activity in the hemolymph and integument (Figure 2A, 2B). However, during the early stages of topically-applied fungal infection, the cuticular PO activity in infected S, but not infected NS larvae, became elevated above uninfected larval levels at 24 h post inoculation (pi) for both *B. bassiana* and *M. anisopliae* infections (P<0.05, P<0.001 Figure 2A), with this being more pronounced in *M. anisopliae* treated insects. This coincides with peak fungal germination and penetration activities.

In contrast, hemolymph PO activity was only elevated significantly in NS larvae exposed to *M. anisopliae* (P<0.01 Figure 2B) while *B. bassiana* failed to trigger significant changes in PO activity in either line relative to the uninfected controls (Figure 2B). Lysozyme-like activity was unchanged in all samples, irrespective of the insect line or treatment (Figure 2C).

An elevated capacity for encapsulation was observed in uninfected S larvae compared with the NS line (P<0.05; Figure 2D), evidenced by a strong melanotic encapsulation of the nylon implant. However, this activity was significantly lower 24 h pi with *B. bassiana* for both lines (Figure 2D; 1.16 times for S line P<0.01; and 1.30 times for the NS line larvae P<0.05 relative to the uninfected controls). Unlike *B. bassiana*, infection by *M. anisopliae* did not lead to changes in the encapsulation response in either line (Figure 2D).

### Immunity- and Stress-related Gene Expression after Topical Infection

**Figure 3:**
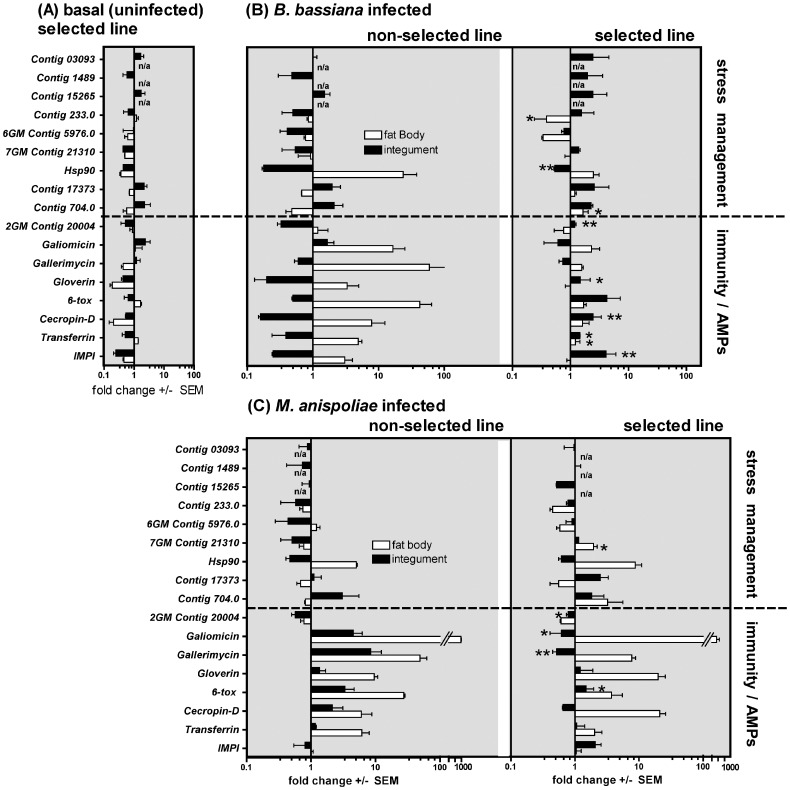
Defense genes expression in integuments and fat body of insects selected by *B. bassiana*. Expression of antimicrobial peptide genes and other putative immunity/stress-management genes in the integument and fat body of non-selected (NS) and selected line (S) larvae, under basal (uninfected) conditions (A), 24 h after topical *B. bassiana* infection (B) and 24 h after *M. anisopliae* topical infection (C). Basal expression in uninfected S larvae (A) is illustrated as a fold change relative to NS uninfected larvae and the x-axis represents basal expression in NS larvae (i.e. 1-fold). Fold induction in infected NS larvae is also calculated relative to NS uninfected larvae (B, C). Fold induction in infected S larvae is calculated relative to the S uninfected expression (B, C). The mean ΔΔCt value of 3 independent experiments (each with a minimum of 5 insects per treatment) and the SEM are reported. *-P<0.05, **-P<0.01, compared with the corresponding induced change in NS line insects. Na = not assayed in fat body tissue.

Expression of seventeen immunity and putative stress management genes was investigated in the integument and fat body of uninfected control and fungal infected insects from both the S and NS lines. The expression data were complex, with some genes behaving differently under each experimental parameter. However, several important trends can be reported. The majority of the studied genes were expressed at a lower basal level in uninfected control S larvae compared with the NS larvae, in both the integument and fat body. However, a group of genes coding for four putative stress-management factors (*Contigs 704, 17373*, *15256, 03093*) and one AMP (*Galiomicin*) exhibited different expression patterns, being slightly higher expressed in the integument (but not the fat body) of control S larvae compared with control NS insects (1.5- to 2.5- fold increase; Figure 3A, Figure S1 and S2).

Following topical fungal infection, the pattern of gene expression in the S-line insects infected with *B. bassiana* was strikingly different from the other three groups of infected insects. In the other groups (i.e. NS insects infected with either fungal species, and in S insects infected with *M. ansipoliae*), fat body expression was characterized by strong upregulation of the genes coding for AMPs, transferrin and Hsp90 (with *M. anisopliae* triggering the strongest responses; up to 910-fold in the case of *Galiomicin* and 50-fold for *Gallerimycin*; P<0.0001 compared with uninfected larvae), while genes with putative roles in stress-management exhibited minimal changes or were mildly downregulated in the fat body after infection. The expression of most stress-management and immunity-related genes in the integument of these infected insects was also either unchanged or downregulated, although some AMP genes were upregulated in the integument of *M. anisopliae* infected NS insects. In contrast, in S-line insects infected with *B. bassiana,* seven of the nine putative stress management genes and six of the eight immunity-related genes were preferentially upregulated in the integument (1.5 to 4.3 fold), concomitant with very low or even downregulated expression of the same genes in the fat body (Figure 3B, 3C, Figure S1, S2, S3, S4). The significance of this overall trend was confirmed by mega-analysis (P<0.01; Table S4). Furthermore, differences between gene expression in the two tissue locations was significant, i.e. *B. bassiana* induced significantly higher gene expression in the S-line integument compared with *M. anisopliae* (P<0.05; Table S5).

It should be noted that overall, and irrespective of the infection state of the insect, expression levels of the genes in the S-line fat body was significantly lower than in NS insects (P<0.01, mega-analysis; Table S4). Upregulation of AMP genes (*Gallerimycin, Galiomicin, Gloverin, Cecropin-D, 6-tox*) and *Transferrin* (a siderophore) was 5–10-times higher in fat body of NS and S fungi infected larvae compared with expression in integuments of infected insects (Figure. 3B, 3C).

### Cost of Resistance on Life History Traits

**Figure 4:**
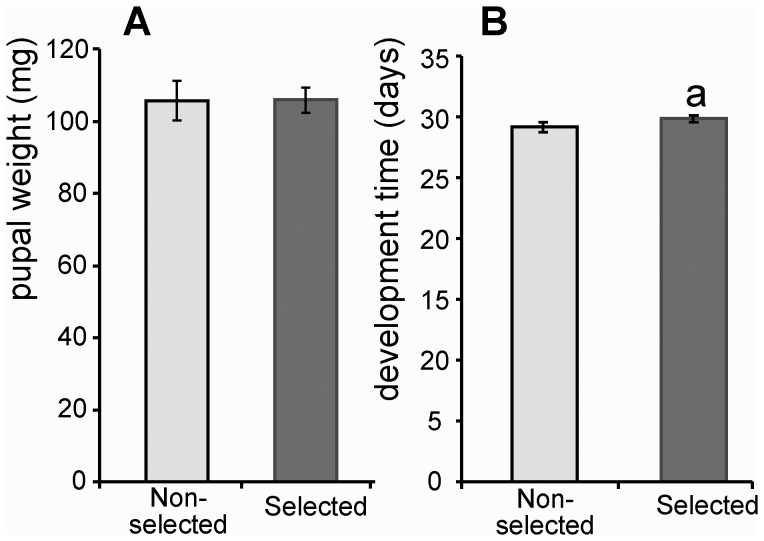
Life-history traits in *G. mellonella* non-selected and selected lines. Pupal weights (A) *n* = at least 52. Larval development time (B) (from egg hatching to onset of pupation) *n* = at least 52 (data presented as mean +/− SEM). a-P<0.05 compared with the NS insects.

Selected insects with enhanced resistance to *B. bassiana* did not display differences in pupal biomass, but larval development time was slightly increased (P<0.05) in S line insects compared with the NS line (Figure 4A, 4B). Both NS and S lines exhibited reduced fecundity (as a consequence of melanism) but this was at equivalent levels in both lines (summarized in Table S2).

## Discussion

The present study shows that insects can develop resistance to insect pathogenic fungi but the resistance is not absolute and is at a cost. A melanic morph of *G. mellonella* was found to have increased resistance to the insect pathogen *B. bassiana* following exposure of successive generations to this fungus. A combination of mechanisms was identified that can account for this resistance, some of which were specific for *B. bassiana* and others which were non-specific antifungal defenses. Both the S (resistant) and NS (susceptible) lines of this morph expressed a wide repertoire of inducible immune and putative stress management genes. In the S line these resources were re-focussed to the integument. These “front line” defenses were only activated by *B. bassiana*.

By concentrating its energies to the first and most important barrier to infection, the host delayed pathogenesis by *B. bassiana* but not *M. anisopliae,* demonstrating a lack of cross-resistance. Similarly, infected S line insects exhibited higher cuticular PO activity but not plasma PO or lysozyme. It is possible the S line had evolved an enhanced *Beauveria* recognition apparatus allowing for a more coordinated and targeted response which would account for the increased activity in the integument but subdued activity in the fat body, the main hemopoietic organ. The fat body occupies a large portion of the insect hemocoel and is the principle site for the synthesis of AMPs in insects exposed to immunogens, including pathogens, irrespective of point of contact or entry [32], [33], [34]. As far as we are aware, there are to date no reports of pathogen specific defense mechanisms involving re-allocation of systemic resources to localised tissues under or at risk of attack.

An insect’s repair and stress pathways limit the damage inflicted by either pathogenesis and/or the host’s own immune response, but little is understood about the relative contribution of damage to the outcome of pathogenesis and the mechanistic links between the immune system and remedial pathways [35], [36]. This study shows elevated integumental expression of all but two of the examined putative stress management genes (i.e. 80%) in the S line in response to *Beauveria* but not *Metarhizium*. The fact that so many putative stress related genes were upregulated signifies their importance in defense responses yet their role is poorly understood and often overlooked. By working in concert with the AMPs they probably mitigate damage and initiate repair resulting in the increased resistance observed in the S line to *B. bassiana.* The importance of the putative stress-response genes is further emphasized in the *M. anisopliae* infected NS insects where upregulation of the AMPs without concomitant upregulation of the stress management genes failed to confer any resistance to the pathogen.

The current study suggests that the resistance in the S line is heritable and multi-factorial, comprising several different physiological traits prioritised not only in terms of location but also timing as they were activated concomitant with the period of fungal penetration of the integument. For example, the elevated cuticular PO activity would inhibit fungal growth through synthesis of melanin and its precursors and through melanin partially shielding cuticular proteins from degradation by Pr1, a major cuticle degrading protease and virulence determinant produced by *B. bassiana* and *M. anisopliae* during the infection process. Elevated expression of IMPI specifically induced by *B. bassiana* in the S line could inhibit metalloproteases produced by insect pathogenic fungi during the infection process [37], [38], [39], [40]. Genes coding for antioxidants are also highly represented under the same conditions and may defend the host against reactive oxygen species generated by PO activity during cuticle penetration [41]. Interestingly, cecropins do have antifungal activity but have been reported as ineffective against *B. bassiana* in other experimental insect systems (e.g. [8]). Current knowledge is very limited regarding the role in *G. mellonella* of 6–tox, although related X-tox AMPs are considered to primarily perform opsonisation roles [42]. Gloverin is not currently known to have any potent antifungal activity, however, putative antifungal and antimicrobial peptides may fulfil different and currently unrecognized roles in different hosts and under specific infection conditions. In isolation, each of these responses are unlikely to sufficiently impart the observed resistance, however, their impact may be amplified by a synergy between these and other as yet unidentified traits.

There is growing evidence that insects can acquire long-term protection against pathogens through immune priming or transfer from the parent to the offspring, a phenomenon referred to as transgenerational immune priming [43], [44]. A wide range of immunogens including pathogens enhance the host’s immune system conferring greater resistance to subsequent exposure to pathogens but the underlying mechanisms of the response remains unclear [45]. The current study shows the 25^th^ generation S line larvae exhibit specific, enhanced resistance to *B. bassiana* without prior exposure to the fungus, suggesting the resistance may be due to specific transgenerational immune priming but with an unexpected degree of specificity and complexity. An earlier but weaker resistance was also observed in the S line which suggests a heritable and amplified immunocompetence. These observations warrant further investigations of possible underlying genetic and epigenetic mechanisms of resistance.

Melanism is strongly correlated with general pathogen resistance with this trait often being accompanied by a trade off in fecundity, development time and even expression of selected immune components such as lysozyme [22], [23], [29], [46]. In the current study, the pupal weight of the melanic S and NS lines (which is directly correlated with fecundity) was similar and already pushed close to minimum. Therefore, the additional drain on resources required to boost antifungal defenses still further, comes not from compromising this life history trait but mainly via a re-allocation of the insect’s immune defenses. It could be argued that resistance in S line insects would increase further with time but to meet the increased demand on resources may result in untenable sacrifices. This is in marked contrast with insects developing resistance to synthetic chemical insecticides where a slight change can have a profound effect [47], [48], [49] whereas resistance to *B. bassiana* clearly involved multiple array of inter-dependent traits. Increased insect resistance to a strain of *B. bassiana* is not a major threat to the use of insect pathogenic fungi as biocontrol agents for several reasons. Firstly, we show that there is no cross resistance to other fungi so the extra investment in defense offers no benefit against other pathogens (introduced or natural). Secondly, the investment in defense is at the expense of fecundity. Thirdly, the downregulation of the AMPs will probably predispose the insect to opportunistic microbial pathogens.

In conclusion, this work reports a previously overlooked adaptation strategy of an insect to a widespread, natural pathogenic fungus. Suppression of systemic responses allows for re-allocation and concentration of resources to the integumental “front line” defenses with an array of immune and stress management factors. This directional selection with *B. bassiana* was specific to this pathogen and not *M. anisopliae.* However, the less fecund insects are probably at no evolutionary advantage in the wild, and we postulate that the risk is small of fungal biological control agents failing in the field.

## Materials and Methods

### Insects

For artificial selection we used insects from a laboratory population of a melanic morph of the Greater wax moth, *Galleria mellonella,* from the Institute of Systematics and Ecology of Animals SB RAS. The starting population was separated into two lines: one line was exposed to the insect pathogenic fungus *Beauveria bassiana* and selected for increased resistance to the pathogen (S line), and the other line was the non-selected untreated control (NS line). The pertinent phenotypic attributes of these lines are summarized in Table S2. The defense responses of the 25^th^ generation of the S and NS insects to *B. bassiana* were compared to elucidate the resistance mechanism(s). Full details of insect rearing and selection are provided in the Text S1.

### Fungal Infections

The susceptibility of S and NS lines to *B*. *bassiana* isolate Sar-31 was determined by natural topical application of conidia. Unlike the previous generations, insects from the 25^th^ generation were not exposed to fungi until they were used for these experiments. Each insect was dipped in an aqueous suspension of the pathogen for 10 s using a concentration of 7.5×10^7^ conidia/ml. Dipped insects were kept in Petri dishes (10 larvae/dish) until sacrificed. The uninfected control insects were dipped into distilled water (n = 60). To determine if there was cross-resistance to other species of fungal pathogen, repeat assays were performed as above, using the insect pathogenic fungus *Metarhizium anisopliae* isolate P-72. Larvae were observed daily for 10 days (up to pupation) for both *B. bassiana* and *M. anisopliae* infections. The emergent adults were monitored for several days to see what percentage were infected with the pathogen. All dead insects were removed and examined to confirm the cause of death. Both fungal isolates were from the Institute of Systematics and Ecology of Animals SB RAS culture collection. All insects used in these experiments were 6^th^ instar larvae raised in the same cohort. The experiment was repeated independently three times. The total number of individuals used from each line was 390 for the *B. bassiana* experiment and 285 for *M. anisopliae*.

### Phenoloxidase Activity in Plasma and Cuticle

Larvae were topically infected with the fungal pathogens as described above, and at 24 post-inoculation (pi), cell-free hemolymph plasma samples and homogenized integuments were prepared for spectrophotometric analysis of phenoloxidase enzymatic activity using L-DOPA as a substrate, and expressed as a change in absorbance/min/mg protein. Uninfected insects were used as a control. The experiment was repeated independently three times. Full details are provided in the Text S1.

### Encapsulation Response

To determine melanotic encapsulation responses to fungal infection, encapsulation assays were performed in NS and S larvae. Nylon monofilament implants were retrieved from the hemocoel and examined using image analysis software to quantify the extent of melanization. Full details are provided in the Text S1 and experiments were repeated independently three times.

### Plasma Lysozyme-like Activity

Antibacterial activity in hemolymph plasma was determined by a zone-of-clearance assay using freeze-dried *Mirococcus lysodeikticus* as a substrate suspended in agarose. The radius of the digested zone was compared with a standard curve made with Egg White Lysozyme [50] and expressed as an EWL equivalent (mg/ml). The experiment was repeated independently three times. Full details are provided in the Text S1.

### QRT-PCR Analysis of Insect Immunity-related Gene Expression

The expression of a range of *G. mellonella* immunity-related genes was quantified in fat body and integument samples dissected from S and NS larvae at 24 h after topical infection with *B. bassiana* or *M. anisopliae*. Gene expression was measured by real-time quantitative RT-PCR using normalised cDNA samples with the Rotor-Gene 6000 (Corbett Research), with Rotor-Gene SYBR Green PCR mix (Qiagen), relative to two reference genes, *18S rRNA* (AF286298) and *Elongation Factor 1-alpha* (EF1; AF423811). Seventeen target genes were investigated, coding for the antimicrobial peptides gallerimycin, galiomicin, gloverin, cecropin D and 6-tox, the siderophore transferrin, the insect metalloproteinase inhibitor (IMPI), one linked to immune signaling (Contig 20004), three coding for heat-shock proteins (HSP-90, contig 21310 and 1489) whose activities ameliorate stress [51], two coding for enzymes dealing with oxidative stress (Contigs 17373 and 03093), one linked to G-protein coupled receptor activity and stress response (Contigs 15265), one involved in anti-apoptosis activity (Contig 5976) and two involved with cell proliferation (Contigs 704 and 233). Full details are provided in the Text S1 and Table S3.

### Life History Traits

The following life history traits were monitored in NS and S insects: larval development time (from egg hatching to onset of pupation) and pupal weight.

### Data Analyses

Data analyses were performed using GraphPad Prism v4.0 (GraphPad Software, USA) and Statistic v6.0 (StatSoft Inc., USA). Data were checked for normal (Gaussian) distribution using the Agostino-Pearson omnibus test, and if abnormally distributed a more conservative non-parametric analysis was applied. In Q-RT-PCR data with a Gaussian distribution, Grubbs’ extreme studentized deviate (ESD) test was used to exclude extreme outliers. In order to assess overall trends associated with selection on basal and induced gene expression, the data from three independently repeated experiments were pooled for mega-analysis after confirming (by 2-way ANOVA) that “experiment”, as treated as a variable, had no significant effect on the outcome. Individual gene comparisons were made with non-parametric one-way ANOVA (Kruskall-Wallis with Dunn’s post test). Cox’s proportional hazards survival regression was used to quantify differences in mortality rates after fungal infections between NS and S larvae. Larvae from bioassay experiment uninfected with the fungi all survived the duration of the experiment, for this reason, the uninfected treatment was ignored in the statistical analysis. One-way ANOVA (Kruskall-Wallis with Dunn’s post test) was used to assess differences between lysozyme, PO and encapsulation responses in S and NS insects. Differences in life history traits were compared by non-parametric t-test (Mann-Whitney). Differences between NS and S larvae, or between treated and control samples, were considered significant when P<0.05.

## Supporting Information

Figure S1 **AMP gene expression in integuments of infected insects.** Expression of antimicrobial peptide genes and other immunity genes in the integument of non-selected (NS) and selected line (S) larvae after topical *B. bassiana* (Bb) and *M. anisopliae* (Ma) infection. Expression of genes was assayed in integument tissue by Q-PCR in uninfected insects, and in insects at 24 h after topical infection. Basal expression in uninfected S larvae (bar 1) is calculated as a fold change relative to NS uninfected larvae. Fold induction in NS larvae infected with Bb (bar 2) and Ma (bar 5) is also calculated relative to NS uninfected larvae. Fold induction in S larvae infected with Bb and Ma is calculated both relative to the S uninfected expression (bars 3 & 6) and relative to the NS uninfected baseline to indicate overall expression (bars 4 & 7). The mean ΔΔCt values of 3 independent experiments are reported +/−95% CI. a-P<0.05, b-P<0.01, compared with fold induction in NS infected same fungus (i.e. comparing S vs NS); c-P<0.05 compared with fold induction in the same line infected by Bb (i.e. comparing Bb vs Ma).
(TIF)

Figure S2 **Stress-management gene expression in integuments of infected insects.** Expression of putative stress-management genes in the integument of non-selected (NS) and selected line (S) larvae after topical *B. bassiana* (Bb) and *M.anisopliae* (Ma) infection. Gene expression was assayed in integument tissue by Q-PCR in uninfected animals, and in animals at 24 h after topical infection. Basal expression in uninfected S larvae (bar 1) is calculated as a fold change relative to NS uninfected larvae. Fold induction in NS larvae infected with Bb (bar 2) and Ma (bar 5) is also calculated relative to NS uninfected larvae. Fold induction in S larvae infected with Bb and Ma is calculated both relative to the S uninfected expression (bars 3 & 6) and relative to the NS uninfected baseline to indicate overall expression (bars 4 & 7) The mean ΔΔCt value of 3 independent experiments the +/−95% CI are reported. a-P<0.05, b-P<0.01, compared with fold induction in NS infected same fungus (i.e. comparing S vs NS); c-P<0.05 compared with fold induction in the same line infected by Bb (i.e. comparing Bb vs Ma).
(TIF)

Figure S3 **AMP gene expression in fat body of infected insects.** Expression of antimicrobial peptide genes in fat body of non-selected (NS) and selected line (S) larvae after topical *B. bassiana* (Bb) and *M.anisopliae* (Ma) infection. Expression of genes was assayed in fat body tissue by Q-PCR in uninfected animals, and in animals at 24 h after topical infections. Basal expression in uninfected S larvae (bar 1) is calculated as a fold change relative to NS uninfected larvae. Fold induction in NS larvae infected with Bb (bar 2) and Ma (bar 5) is also calculated relative to NS uninfected larvae. Fold induction in S larvae infected with Bb and Ma is calculated both relative to the S uninfected expression (bars 3 & 6) and relative to the NS uninfected baseline to indicate overall expression (bars 4 & 7). The mean ΔΔCt values of 3 independent experiments are reported +/−95% CI. a-P<0.05, b-P<0.01, compared with fold induction in NS infected same fungus (i.e. comparing S vs NS); c-P<0.05 compared with fold induction in the same line infected by Bb (i.e. comparing Bb vs Ma).
(TIF)

Figure S4 **Stress-management gene expression in fat body of insects.** Expression of putative stress-management genes in fat body of non-selected (NS) and selected line (S) larvae after topical *B. bassiana* (Bb) and *M.anisopliae* (Ma) infection. Expression of genes was assayed in fat body tissue by Q-PCR in uninfected animals, and in animals at 24 h after topical infections. Basal expression in uninfected S larvae (bar 1) is calculated as a fold change relative to NS uninfected larvae. Fold induction in NS larvae infected with Bb (bar 2) and Ma (bar 5) is also calculated relative to NS uninfected larvae. Fold induction in S larvae infected with Bb and Ma is calculated both relative to the S uninfected expression (bars 3 & 6) and relative to the NS uninfected baseline to indicate overall expression (bars 4 & 7). The mean ΔΔCt values of 3 independent experiments are reported +/−95% CI. a-P<0.05, b-P<0.01, compared with fold induction in NS infected same fungus (i.e. comparing S vs NS); c-P<0.05 compared with fold induction in the same line infected by Bb (i.e. comparing Bb vs Ma).
(TIF)

Table S1 Susceptibility of *G. mellonella* selected and non-selected lines to *B. bassiana*. Susceptibility of *Galleria mellonella* larvae of selected and non-selected lines to topical fungal infection with *Beauveria bassiana* (7.5×10^7^ conidia/ml).
(DOC)

Table S2 Attributes of melanic and non-melanic *G. mellonella*. Attributes of selected (resistant) and non-selected (susceptible) melanic morphs of 5th instar *Galleria mellonella* larvae compared with a non-melanic morph.
(DOC)

Table S3 Loci used for expression analysis.
(XLS)

Table S4 Mega-analysis of Q-PCR data. Summary showing trends in gene expression in S and NS line *G. mellonella* larvae in different tissues following infection with *B. bassiana* and *M. anispoliae*: effect of selection on gene expression.
(DOC)

Table S5 Mega-analysis of Q-PCR data. Summary showing trends in gene expression in S and NS line *G. mellonella* larvae in different tissues following infection with *B. bassiana* and *M. anispoliae*: effect of fungal species on gene expression.
(DOC)

Text S1 **Referenced experimental procedures.**
(DOC)
