# Correction: Can Insects Develop Resistance to Insect Pathogenic Fungi?

**DOI:** 10.1371/annotation/3c61c1d6-7981-4f3e-a690-1ce7a4d89285

**Published:** 2014-01-13

**Authors:** Ivan M. Dubovskiy, Miranda M. A. Whitten, Olga N. Yaroslavtseva, Carolyn Greig, Vadim Y. Kryukov, Ekaterina V. Grizanova, Krishnendu Mukherjee, Andreas Vilcinskas, Viktor V. Glupov, Tariq M. Butt

The name of a funding organization for the first, second and last authors is incorrect. The Funding statement should read: 

"Funding from both the Royal Society International Joint Project and the Russian Foundation for Basic Research (awarded to TB, MW, ID), presidium SBRAS (VG, ID, VK) and the DFG Priority Program 1399 'Host-Parasite-Coevolution – rapid reciprocal adaptation and its genetic basis' to AV (VI 219/3-1). The funders had no role in study design, data collection and analysis, decision to publish, or preparation of the manuscript."

