# Supplementary figures and images for: Can Insects Develop Resistance to Insect Pathogenic Fungi?
Source: PLoS One. 2013 Apr 1;8(4):e60248. doi: 10.1371/journal.pone.0060248 (PMC3613352; doi:10.1371/journal.pone.0060248)

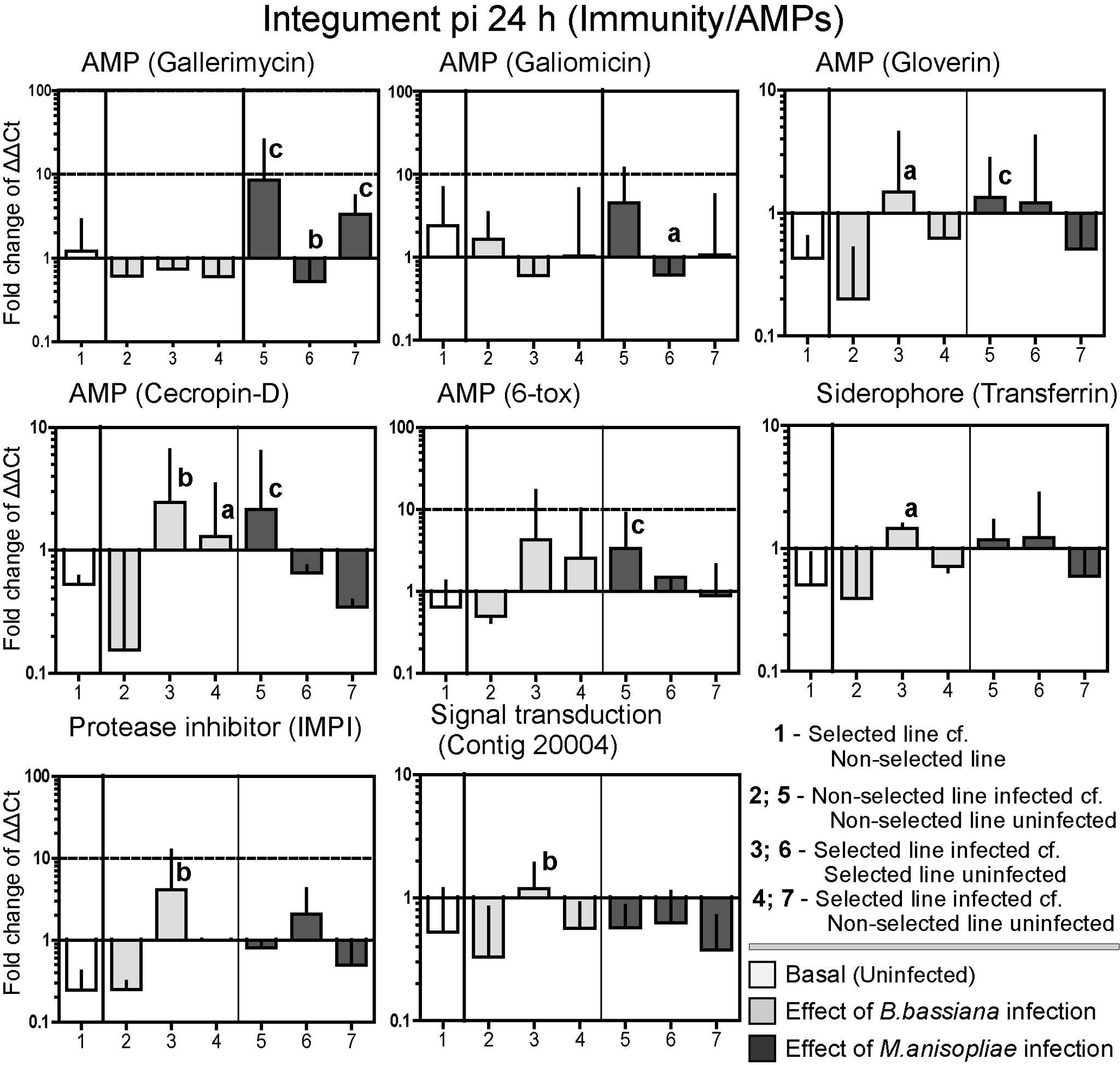

Supplement: Figure S1 — AMP gene expression in integuments of infected insects. Expression of antimicrobial peptide genes and other immunity genes in the integument of non-selected (NS) and selected line (S) larvae after topical B. bassiana (Bb) and M. anisopliae (Ma) infection. Expression of genes was assayed in integument tissue by Q-PCR in uninfected insects, and in insects at 24 h after topical infection. Basal expression in uninfected S larvae (bar 1) is calculated as a fold change relative to NS uninfected larvae. Fold induction in NS larvae infected with Bb (bar 2) and Ma (bar 5) is also calculated relative to NS uninfected larvae. Fold induction in S larvae infected with Bb and Ma is calculated both relative to the S uninfected expression (bars 3 & 6) and relative to the NS uninfected baseline to indicate overall expression (bars 4 & 7). The mean ΔΔCt values of 3 independent experiments are reported +/−95% CI. a-P<0.05, b-P<0.01, compared with fold induction in NS infected same fungus (i.e. comparing S vs NS); c-P<0.05 compared with fold induction in the same line infected by Bb (i.e. comparing Bb vs Ma). (TIF) [file pone.0060248.s001.tif]

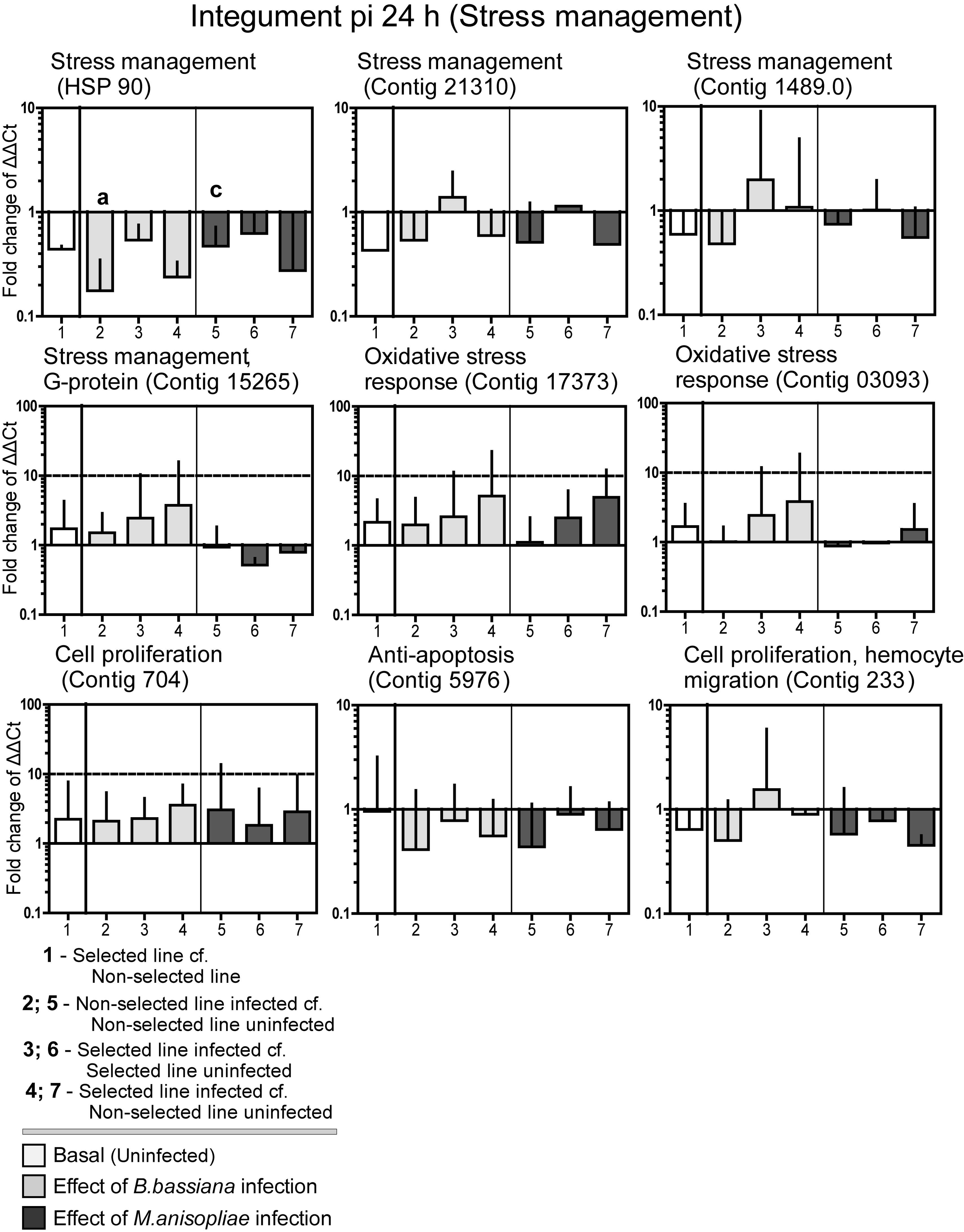

Supplement: Figure S2 — Stress-management gene expression in integuments of infected insects. Expression of putative stress-management genes in the integument of non-selected (NS) and selected line (S) larvae after topical B. bassiana (Bb) and M.anisopliae (Ma) infection. Gene expression was assayed in integument tissue by Q-PCR in uninfected animals, and in animals at 24 h after topical infection. Basal expression in uninfected S larvae (bar 1) is calculated as a fold change relative to NS uninfected larvae. Fold induction in NS larvae infected with Bb (bar 2) and Ma (bar 5) is also calculated relative to NS uninfected larvae. Fold induction in S larvae infected with Bb and Ma is calculated both relative to the S uninfected expression (bars 3 & 6) and relative to the NS uninfected baseline to indicate overall expression (bars 4 & 7) The mean ΔΔCt value of 3 independent experiments the +/−95% CI are reported. a-P<0.05, b-P<0.01, compared with fold induction in NS infected same fungus (i.e. comparing S vs NS); c-P<0.05 compared with fold induction in the same line infected by Bb (i.e. comparing Bb vs Ma). (TIF) [file pone.0060248.s002.tif]

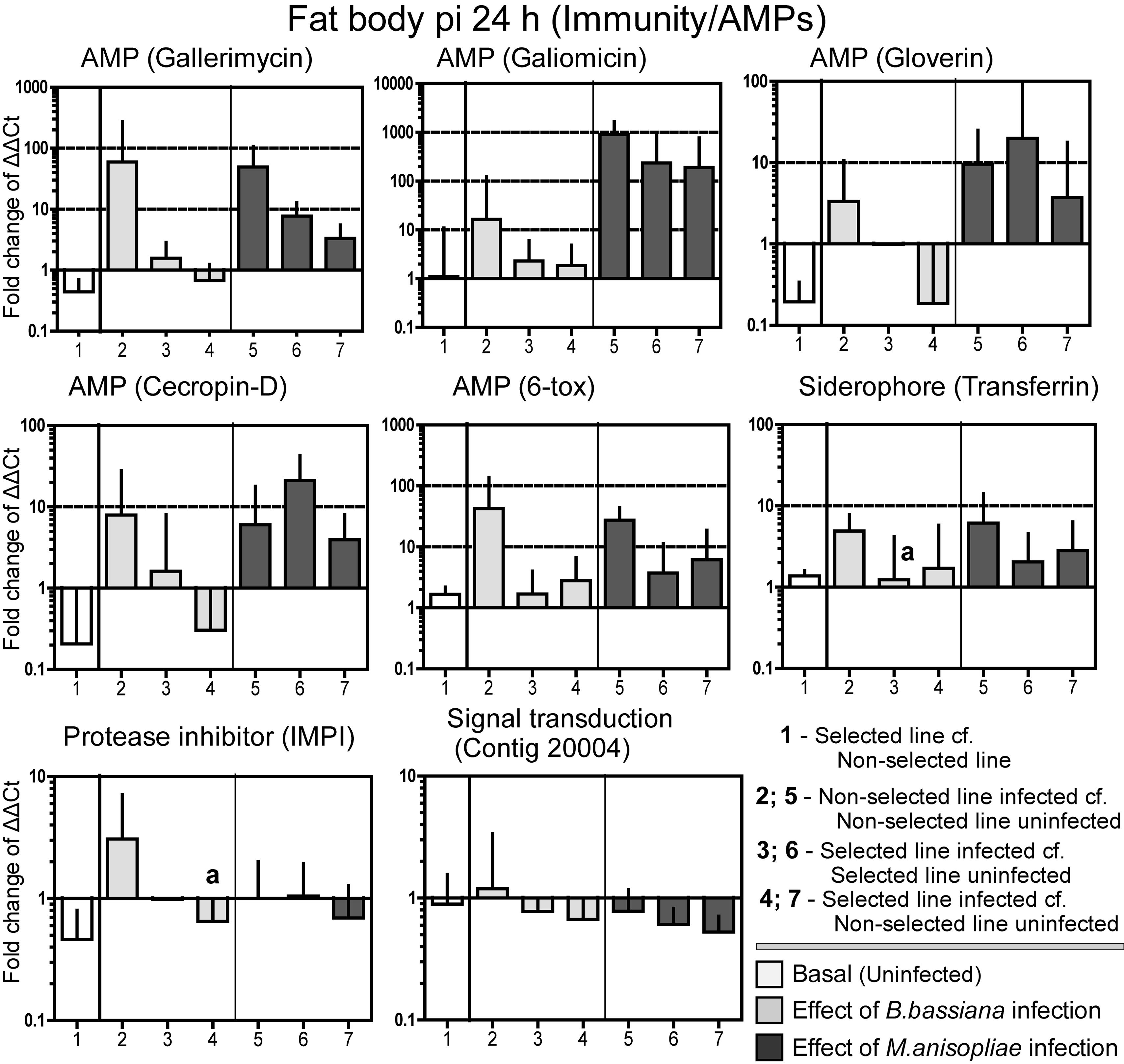

Supplement: Figure S3 — AMP gene expression in fat body of infected insects. Expression of antimicrobial peptide genes in fat body of non-selected (NS) and selected line (S) larvae after topical B. bassiana (Bb) and M.anisopliae (Ma) infection. Expression of genes was assayed in fat body tissue by Q-PCR in uninfected animals, and in animals at 24 h after topical infections. Basal expression in uninfected S larvae (bar 1) is calculated as a fold change relative to NS uninfected larvae. Fold induction in NS larvae infected with Bb (bar 2) and Ma (bar 5) is also calculated relative to NS uninfected larvae. Fold induction in S larvae infected with Bb and Ma is calculated both relative to the S uninfected expression (bars 3 & 6) and relative to the NS uninfected baseline to indicate overall expression (bars 4 & 7). The mean ΔΔCt values of 3 independent experiments are reported +/−95% CI. a-P<0.05, b-P<0.01, compared with fold induction in NS infected same fungus (i.e. comparing S vs NS); c-P<0.05 compared with fold induction in the same line infected by Bb (i.e. comparing Bb vs Ma). (TIF) [file pone.0060248.s003.tif]

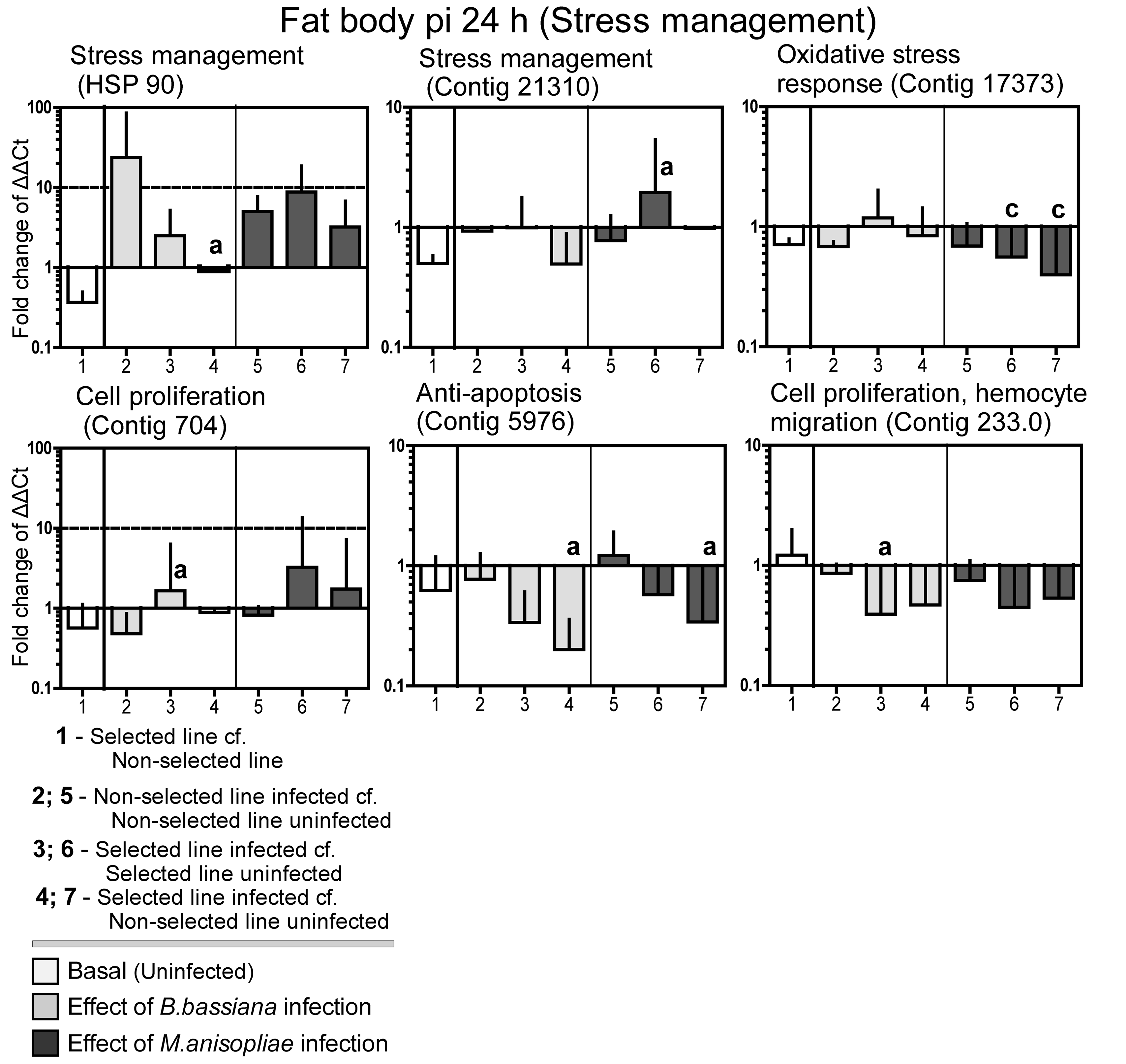

Supplement: Figure S4 — Stress-management gene expression in fat body of insects. Expression of putative stress-management genes in fat body of non-selected (NS) and selected line (S) larvae after topical B. bassiana (Bb) and M.anisopliae (Ma) infection. Expression of genes was assayed in fat body tissue by Q-PCR in uninfected animals, and in animals at 24 h after topical infections. Basal expression in uninfected S larvae (bar 1) is calculated as a fold change relative to NS uninfected larvae. Fold induction in NS larvae infected with Bb (bar 2) and Ma (bar 5) is also calculated relative to NS uninfected larvae. Fold induction in S larvae infected with Bb and Ma is calculated both relative to the S uninfected expression (bars 3 & 6) and relative to the NS uninfected baseline to indicate overall expression (bars 4 & 7). The mean ΔΔCt values of 3 independent experiments are reported +/−95% CI. a-P<0.05, b-P<0.01, compared with fold induction in NS infected same fungus (i.e. comparing S vs NS); c-P<0.05 compared with fold induction in the same line infected by Bb (i.e. comparing Bb vs Ma). (TIF) [file pone.0060248.s004.tif]
