# Supplementary material for: Can Insects Develop Resistance to Insect Pathogenic Fungi?
Source: PLoS One. 2013 Apr 1;8(4):e60248. doi: 10.1371/journal.pone.0060248 (PMC3613352; doi:10.1371/journal.pone.0060248)
Supplement: Table S1 — Susceptibility of G. mellonella selected and non-selected lines to B. bassiana. Susceptibility of Galleria mellonella larvae of selected and non-selected lines to topical fungal infection with Beauveria bassiana (7.5×10^7^ conidia/ml). (DOC) [file pone.0060248.s005.doc]

**Table S1**

Susceptibility of *Galleria mellonella* larvae of selected and non-selected lines to topical fungal infection with *Beauveria bassiana* (7.5x10^7^ conidia / ml)

| **F** | **Line** | **LT25, day** | **LT50, day** | **LT75, day** | **Total death, 10 day, %** | **n** | **p** |
| --- | --- | --- | --- | --- | --- | --- | --- |
| **5** | Selected | 6 | 7 | 8 | 84 | 153 | 0,229 |
|  | Non-selected | 5 | 6 | 7 | 88 | 174 |  |
| **10** | Selected | 5 | 6 | 9 | 86 | 187 | 0.333 |
|  | Non-selected | 5 | 6 | 9 | 87 | 189 |  |
| **20** | Selected | 6 | 7 | 8 | 86 | 150 | 0.143 |
|  | Non-selected | 5 | 6 | 7 | 88 | 170 |  |
| **25** | Selected | 5 | 6 | 8 | 79 | 196 | 0.010 |
|  | Non-selected | 5 | 6 | 7 | 89 | 195 |  |
