# Supplementary material for: Can Insects Develop Resistance to Insect Pathogenic Fungi?
Source: PLoS One. 2013 Apr 1;8(4):e60248. doi: 10.1371/journal.pone.0060248 (PMC3613352; doi:10.1371/journal.pone.0060248)
Supplement: Table S2 — Attributes of melanic and non-melanic G. mellonella. Attributes of selected (resistant) and non-selected (susceptible) melanic morphs of 5th instar Galleria mellonella larvae compared with a non-melanic morph. (DOC) [file pone.0060248.s006.doc]

**Table S2**

Attributes of selected (resistant) and non-selected (susceptible) melanic morphs of 5th instar *Galleria mellonella* larvae compared with a non-melanic morph

| **Attribute** | **Melanic morph** | **Melanic morph** | **Non-melanic morph** |
| --- | --- | --- | --- |
|  | **Non-Selected (NS)** | **Selected (S)** | (for comparison *) |
| Appearance of cuticle | Dark, thick | Dark, thick | Pale, thin |
| Susceptibility to insect pathogenic fungus *Beauveria bassiana* | Moderate | Low | High |
| Susceptibility to insect pathogenic fungus *Metarhizium anisopliae* | Moderate | Moderate | High |
| Pupal biomass | Reduced | Reduced | Normal |
| Adult fecundity (eggs laid per female) | Low | Low | High |

* The non-melanic morph was not used in current study, but is shown here for comparison (all the attributes were assessed by the authors).
