# Supplementary material for: Can Insects Develop Resistance to Insect Pathogenic Fungi?
Source: PLoS One. 2013 Apr 1;8(4):e60248. doi: 10.1371/journal.pone.0060248 (PMC3613352; doi:10.1371/journal.pone.0060248)
Supplement: Table S4 — Mega-analysis of Q-PCR data. Summary showing trends in gene expression in S and NS line G. mellonella larvae in different tissues following infection with B. bassiana and M. anispoliae: effect of selection on gene expression. (DOC) [file pone.0060248.s008.doc]

**Table S4 Mega-analysis of Q-PCR data.**

Summary showing trends in gene expression in S and NS line *G. mellonella* larvae in different tissues following infection with *B. bassiana* and *M. anispoliae*: effect of selection on gene expression.

|  |  | **basal expression** | | **infected with B. bassiana** | | **infected with M. anisopliae** | |
| --- | --- | --- | --- | --- | --- | --- | --- |
|  |  | **Fat Body** | **Cuticle** | **Fat Body** | **Cuticle** | **Fat Body** | **Cuticle** |
| **Is the influence of selection significant?** | **overall (all genes)** | Selection has a significant impact on overall expression levels P<0.01 (lower expression in S insects) | Selection does not significantly affect expression levels | Selection has a significant impact on overall expression levels P<0.01 (lower expression in S insects) | Selection has a significant impact on overall expression levels P<0.01 (higher expression in S insects) | Selection has a significant impact on overall expression levels P<0.01 (lower expression in S insects) | Selection does not significantly affect expression levels |
|  | **AMP & immunity-related genes** | Selection does not significantly affect expression levels | Selection does not significantly affect expression levels | Selection does not significantly affect expression levels | Selection has a significant impact on overall expression levels P<0.01 (higher expression in S insects) | Selection has a significant impact on expression levels P<0.01 (lower expression in S insects) | Selection has a significant impact on expression levels P<0.05 (lower expression in S insects) |
|  | **putative stress-management genes** | Selection does not significantly affect expression levels | Selection does not significantly affect expression levels | Selection does not significantly affect expression levels | Selection does not significantly affect expression levels | Selection does not significantly affect expression levels | Selection does not significantly affect expression levels |
