# Supplementary material for: Can Insects Develop Resistance to Insect Pathogenic Fungi?
Source: PLoS One. 2013 Apr 1;8(4):e60248. doi: 10.1371/journal.pone.0060248 (PMC3613352; doi:10.1371/journal.pone.0060248)
Supplement: Table S5 — Mega-analysis of Q-PCR data. Summary showing trends in gene expression in S and NS line G. mellonella larvae in different tissues following infection with B. bassiana and M. anispoliae: effect of fungal species on gene expression. (DOC) [file pone.0060248.s009.doc]

**Table S5 Mega-analysis of Q-PCR data**. Summary showing trends in gene expression in S and NS line *G. mellonella* larvae in different tissues following infection with *B. bassiana* and *M. anispoliae*: effect of fungal species on gene expression.

|  | **Selected Line** | | **Non-selected Line** | |
| --- | --- | --- | --- | --- |
|  | **fatbody** | **cuticle** | **fatbody** | **cuticle** |
| Do the tested genes respond in the same way to both fungal species in each insect line? | no significant effect on overall gene expression | *B. bassiana* triggers significantly higher gene expression than *M. anispoliae* P<0.05 | *M. anispoliae* triggers very significantly higher gene expression than *B. bassiana*. P<0.0001 | *M. anispoliae* triggers significantly higher gene expression than *B. bassiana.* P<0.05 |
