# Supplementary material for: Can Insects Develop Resistance to Insect Pathogenic Fungi?
Source: PLoS One. 2013 Apr 1;8(4):e60248. doi: 10.1371/journal.pone.0060248 (PMC3613352; doi:10.1371/journal.pone.0060248)
Supplement: Text S1 — Referenced experimental procedures. (DOC) [file pone.0060248.s010.doc]

##### Supporting Text S1

##### Experimental Procedures

#### Reagents and buffers

Freeze-dried *Micrococcus lysodeikticus*, egg white lysozyme, bovine serum albumin, phenylthiourea, ethylenediamine tetraacetic acid and 3,4-dihydroxy-L-phenylalanine were all supplied by Sigma–Aldrich, UK. All solutions were made in pyrogen-free ultra-pure water.

**Insect rearing and selection**

The experimental and stock populations of *G. mellonella* were reared in strict isolation at 28°C, 60% relative humidity, with a 12:12h light:dark cycle, and fed on artificial medium (AM) containing 22.5% corn meal, 12.5% honey, 12.5% glycerol, 12.5% beeswax, 10% wheat flour, 12.5% milk solids, 5% yeast and 12.5% water. At the start of the artificial selection procedure, the laboratory insect population was split into two separate lines. Imagoes (100 males; 100 females) were placed in a 10L plastic tank at 28 °C in the dark to lay eggs on AM. One line was then subjected to selection (Selected line, S) and the second was left as the non-selected line (NS). At least 1000 5^th^-6th instar larvae were used for selection for each of the first three generations followed by at least 400 larvae for subsequent selections. In each selection cycle, 400 S-line larvae were infected by 5 x 10^6^ *B. bassiana* conidia to establish a mycosis (LC_40-50_). Fresh fungal conidia (1 week old) were used to infect insects for each round of selection. The NS line insects were always kept under fungus-free conditions. To initiate the S-line next generation, 200 of the surviving larvae were collected and allowed to breed. This number of insects per population is enough to reduce the risks of inbreeding and genetic drift . Two hundred uninfected NS-line insects (of exactly the same age as those used for the S-line) were also collected and allowed to breed. The experiment was continued for 25 generations. Survival assays using *B. bassiana* infection were performed with the 5^th^, 10^th^, 20^th^ and 25^th^ generations of both the S and NS lines (Table S1). At 25 generations, we tested lines (NS and S) for defense reactions under *B. bassiana* and *M. anisopliae* infection conditions, 24 h pi.

**Fungus cultivation**

Fungi were grown on Sabouraud’s dextrose agar (SDA) at 25°C for 14 d. Conidia were harvested by scraping from sporulating cultures, air-dried at RT for 1 week and stored at 4°C. For topical infections, conidia were suspended in sterile 0.03% Tween–80 and vortexed for 1 min. Viability of conidia was verified by incubation of these propagules on SDA and determining the percentage germination. Only suspensions with at least 99% germination were used.

#### Phenoloxidase activity in plasma and cuticle

NS and S larvae were topically infected with fungus as described above, and at 24h post infection, cell-free hemolymph plasma samples and homogenized cuticle fragments were prepared for analysis of phenoloxidase (PO) activity. Hemolymph plasma fractions were prepared by collecting 10 µl of hemolymph from an incision made on the third proleg per larva. This was diluted with 20 µl PBS and centrifuged at 500g for 5 min at 4°C to remove the hemocyte pellet. Whole cleaned integument was dissected in PBS, washed 3 times by vortexing for 1 min in PBS, and homogenized in 200 µl PBS for 2 min at 6.5 M/s with a FASTPREP®-24 homogenizer (MP Biomedicals, USA). The homogenates were centrifuged for 15 min at 12000xg at 4°C. The supernatants of both the hemolymph plasma and the cuticle were then used for spectrophotometric analysis of PO enzymatic activity and protein concentration in a modification of the method described by . Ten microliter aliquots of plasma or 50 µl of cuticle homogenate were added to wells of a flat-bottomed 96-well microtitre plate containing 200 µl of 10 mM L-DOPA (L-3,4-dihyroxyphenylalanine dissolved in sterile pyrogen-free water). After 30 min (for plasma) or 3 h (for cuticle) at 28°C, the absorbance was quantified at 490 nm using a plate reader (BMG Labtech, Germany). The units of PO activity were expressed as the change in absorbance at 490nm per 1 min per mg of protein. The protein concentration of the samples was estimated by the method of Bradford using bovine serum albumin standards.

#### Encapsulation

Hemocytic encapsulation was investigated from freshly collected hemolymph of S and NS larvae, before and 24h after topical fungus application. The strength of the encapsulation response was assayed by implanting a 2 mm long, 0.5 mm diameter piece of white nylon monofilament with nodule on the end (implant). Implants were injected into the hemocoel through a perforation in the ventral segment of the cuticle and nodule was fixative part that prevented implants moving into or out from hemocoel. Implants were dissected out from the body cavity after 2 h, and photographed from three angles. ImageJ 1.45 (National Institute of Health, USA) was used to quantify the extent of melanization of each implant (the “gray value” i.e. the sum of the gray value of all the pixels in the “white balanced” selected area divided by the number of pixels), and these were compared with fresh control implants without melanization .

#### Plasma lysozyme-like activity

A radial diffusion assay was used to determine lysozyme-like antibacterial activity in cell-free hemolymph plasma, as used by . A 10 µl aliquot of hemolymph from each larva was collected into 5 µl ice-cold anticoagulant comprised 62 mM NaCl, 100 mM glucose, 10 mM EDTA, 30 mM sodium citrate and 26 mM citric acid (pH 4.6) with 0.4% PTU and centrifuged for 5 min at 500g, 4°C, to pellet the hemocytes. Four µl cell-free supernatant was then applied to 1.5% agarose in physiological solution (NaCl 0.9%) containing 4 mg / ml freeze-dried *Micrococcus lysodeikticus* (Sigma-Aldrich, UK). The agarose plates were incubated at 28°C for 18 h, and lysozyme-like activity was observed as a clear zone of digested *M. lysodeikticus* lyophilized cells. The radius of clearance was measured and quantiﬁed from a standard curve made with EWL (egg white lysozyme, Sigma-Aldrich, UK) and expressed as an EWL equivalent (mg / ml).

#### Fat body and integument RNA extraction and cDNA synthesis

NS and S larvae were sampled at 24h after topical infection with *B. bassiana* or *M. anisopliae* and corresponding controls. Fat body and integument was dissected from five chilled, surface-sterilized *G. mellonella* larvae per treatment, and stored in RNA-later (Ambion) then freeze-dried and crushed in liquid nitrogen. Total RNA was isolated using TRIzol® Reagent (Invitrogen) according to the manufacturer’s recommendations. RNA concentrations were determined spectrophometrically, and 2μg RNA were treated with deoxyribonuclease I (Promega), at 37°C for 30 min. cDNA synthesis was performed with 1μg RNA using qScript™ cDNA SuperMix (Quanta Bioscience). The experiment was repeated three times at time point and with each line of larva, and each of the triplicate samples comprised fat body or integument from five individuals.

**QRT-PCR**

cDNA quantity was checked and normalized using reference gene PCR of 1/50 dilutions of each sample measured against a standard curve, and sufficient cDNA of similar concentration for each sample diluted to amplify all genes. Samples were quality checked for consistency between values for the two reference genes used: *18S rRNA* (AF286298) and *Elongation Factor 1-alpha* (EF1) (AF423811). Expression was then measured in the normalized samples using a Rotor-Gene 6000 (Corbett Research), with Rotor-Gene SYBR Green PCR mix (Qiagen), relative to these two reference genes. Primers were designed from published *G. mellonella*sequences (NCBI) or from coding sequence where high homology protein sequences could be identified from an EST library and are given in Table S2. For *HSP90*, a primer designed to the conserved 3’UTR region found in Lepidoptera was paired with a degenerate primer designed from an alignment of 8 lepidopteran *HSP90* sequences (GU230738, AB214972, AB060275, EF197936, GU230737, AF254880, GU230739, AB206477) using CODEHOP . Other primers were designed using PerlPrimer or Primer3 to amplify at 60˚C with an amplicon size of 80-200bp, rechecked for potential dimer formation with Oligo 6 (Molecular Biology Insights, Inc, http://oligo.net/), and for amplicon secondary structure using the Mfold server . Primers were optimized by checking products for a clean single peak by high resolution melt curve (HRM) analysis and by titrating concentration for optimal efficiency using a serial dilution of mixed cDNA, and were redesigned if necessary to produce a clean PCR product.

A mix of 5 μl Rotor-Gene SYBR Green PCR mix (Qiagen) and 1 μl primer was added to 4 μl cDNA for each 10 μl PCR. Cycling conditions were 95˚C 5 min followed by 42 cycles of: 95˚C 5 sec, annealing 10sec, 72˚C 20 sec. An initial touchdown of 1˚C per cycle from 65˚C for the first 5 cycles. HRM analysis performed at the end of each run allowed each PCR to be checked for the presence of the expected product. All reactions were performed in triplicate, and optimal threshold values and reaction efficiencies calculated from 7-point serial dilutions of mixed cDNA from fungal infected insects. Fold change values were calculated using the ΔΔCt method: for each locus, the ΔΔCt for sample was determined by subtracting the measured Ct value from the Ct value of each reference or ‘housekeeping’ gene. ΔΔCts were then converted to relative copy numbers with the formula 2^Δ^ΔΔ^Ct^. Fold changes were also calculated using reaction efficiencies using the Pffafl equation . Values showed similar trends for both reference genes and for each method of calculation: ΔΔCt values for EF1 are shown.

**REFERENCES FOR SUPPORTING TEXT S1**

1. Roy BA, Kirchner JW (2000) Evolutionary dynamics of pathogen resistance and tolerance. Evolution 54: 51-63.

2. Ashida M, Söderhäll K (1984) The prophenoloxidase activating system in crayfish. Comparative Biochemistry and Physiology Part B: Comparative Biochemistry 77: 21–26.

3. Bradford MM (1976) A rapid and sensitive method for the quantitation of microgram quantities of protein utilizing the principle of protein-dye binding. Analytical Biochemistry 7: 248-254.

4. Rantala MJ, Roff DA (2006) Analysis of the importance of genotypic variation, metabolic rate, morphology, sex and development time on immune function in the cricket, *Gryllus firmus*. Journal of Evolutionary Biology 19: 834-843.

5. Dubovskiy IM, Krukova NA, Glupov VV (2008) Phagocytic activity and encapsulation rate of *Galleria mellonella* larval haemocytes during bacterial infection by *Bacillus thuringiensis*. Journal of Invertebrate Pathology 98: 360-362.

6. Dubovskiy IM, Grizanova EV, Ershova NS, Rantala MJ, Glupov VV (2011) The effects of dietary nickel on the detoxification enzymes, innate immunity and resistance to the fungus *Beauveria bassiana* in the larvae of the greater wax moth *Galleria mellonella*. Chemosphere 85: 92-96.

7. Wojda I, Kowalski P, Jakubowicz T (2009) Humoral immune response of *Galleria mellonella* larvae after infection by *Beauveria bassiana* under optimal and heat-shock conditions. Journal of Insect Physiology 55: 525-531.

8. Vogel H, Altincicek B, Glockner G, Vilcinskas A (2011) A comprehensive transcriptome and immune-gene repertoire of the lepidopteran model host *Galleria mellonella*. BMC Genomics 12: 308.

9. Staheli JP, Boyce R, Kovarik D, Rose TM (2011) CODEHOP PCR and CODEHOP PCR primer design. Methods in Moleclar Biology 687: 57-73.

10. Marshall OJ (2004) PerlPrimer: cross-platform, graphical primer design for standard, bisulphite and real-time PCR. Bioinformatics 20: 471-2472.

11. Rozen S, Skaletsky H (2000) Primer3 on the WWW for general users and for biologist programmers. Methods in Moleclar Biology 132: 365-386.

12. Zuker M (2003) Mfold web server for nucleic acid folding and hybridization prediction. Nucleic Acids Research 31: 3406-3415.

13. Pfaffl MW (2001) A new mathematical model for relative quantification in real-time RT-PCR. Nucleic Acids Research 29: e45.
